# Efficacy and safety of acupuncture for posterior circulation ischemic vertigo: A protocol for systematic review and meta-analysis

**DOI:** 10.1097/MD.0000000000022132

**Published:** 2020-09-11

**Authors:** Bo-xuan Li, Chen Li, Yu-zheng Du, Xiang-gang Meng

**Affiliations:** Acupuncture and Moxibustion Department, National Clinical Research Center for Chinese Medicine Acupuncture and Moxibustion, First Teaching Hospital of Tianjin University of Traditional Chinese Medicine, Changling Road, Xiqing District, Tianjin, China.

**Keywords:** acupuncture, protocol, posterior circulation ischemic vertigo, systematic review

## Abstract

**Background:**

Posterior circulation ischemic vertigo (PCIV) is one of the most complaint symptoms in clinical, and is associated with high risk of recurrence. Current studies show that acupuncture has therapeutic effect on releasing symptom as well as improving the blood flow of posterior circulation. In this review, we aim to assess the efficacy and safety of acupuncture for PCIV.

**Methods:**

Literature of clinical randomized control trials regarding acupuncture for PCIV published before August of 2020 will be searched in databases, including 5 English databases and 4 Chinese databases. For the included studies, methodological quality will be assessed according to Cochrane Risk of Bias Tool, and evidence quality will be evaluated with Grading of Recommendations Assessment, Development and Evaluation guidelines. Data analysis will be performed using Review Manager Software.

**Results:**

The primary outcomes involve changes of PCIV symptoms and blood flow velocity of vertebrobasilar. The secondary outcomes include Barthel Index, National Institute of Health Stroke Scale, clinical effectiveness, and adverse reactions.

**Conclusion:**

Based on current clinical studies, this systematic review and meta-analysis will provide evidence-based basis for the efficacy and safety of acupuncture in treating PCIV.

**Trial registration:**

The protocol for this review has been registered in the INPLASY network (Registration number: INPLASY202070116).

## 1 Introduction

Cerebrovascular disease is one of the leading causes of DALYs all over the world,^[1]^ and 80% are ischemic cerebrovascular disease. Posterior circulation ischemic (POCI) takes account of 20% in ischemic stroke,^[2]^ leaving plenty of poststroke survivors that increases the burden of society.^[3]^ The complex signs and symptoms of POCI complicate the diagnosis and management compared with anterior circulation ischemic,^[4]^ increasing difficulty of early diagnosis. While primary motor and sensory pathways are usually intact in POCI, vertigo becomes a prodrome of it,^[5,6]^ which might be helpful for early diagnosis of stroke. Clinical evidence shows that posterior circulation ischemic vertigo (PCIV) has a high risk of recurrence and is associated with mental disturbance such as anxiety and depression,^[7,8]^ which should be alert by clinicians.

The management of PCIV parallels that of POCI and vertigo,^[9]^ including surgical and pharmacological treatment. But thrombolytic therapy is strictly limited by occurring time and patient's condition,^[10]^ and long-term pharmacological treatment increases patient's mental stress. While neurosurgical intervention has strict limitations, patients that out of therapeutic time window may not get effective treatment.^[11]^ Thus, comprehensive multiple, combined methods are needed, and acupuncture provides an opportunity for PCIV patients as an alternative and complementary treatment.

Acupuncture has been practiced for more than 2000 years and is experienced in treating stroke and vertigo since Han Dynasty. In 1979, the World Health Organization (WHO) recommended it as an alternative and complementary strategy for stroke treatment and rehabilitation, and has been used in many countries such like the USA, UK, Australia, and Scandinavian countries.^[12–15]^ Clinical and laboratory studies show that acupuncture is safe and has therapeutic effect for ischemic stroke and vertigo patients^[16–19]^ Acupuncture reduces discomforts of vertigo and decreases neurological impairment through prompting cerebral blood flow.^[20,21]^ However, systematic review that focuses on efficacy of acupuncture for PCIV is lacked. Based on these, we aim to evaluate the efficacy and safety of acupuncture for PCIV patients.

## 2 Study registration

We have registered this systematic review in INPLASY (Registration number: INPLASY202070116). This study protocol will be reported under the guide of the Preferred Reporting Items for Systematic Reviews and Meta-Analyses (PRISMA) statement.^[22]^ The Cochrane Handbook for Systematic Reviews of Interventions will be used to conduct the study.^[23]^

## 3 Methods

### 3.1 Inclusion criteria

#### 3.1.1 Study type

Randomized controlled trials (RCTs) that related to PCIV will be included irrespective of blinding, publication status, or language. Quasi-RCTs, animal studies, case reports, and reviews will be excluded. Duplicate publications are preferred to the most recent and comprehensive data one.

#### 3.1.2 Participants

Patients who clinically diagnosed with PCIV will be included. According to the diagnostic criteria for PCIV of WHO patients must experience dizziness or vertigo that may be accompanied with neurological deficiency.^[24]^ The imaging evidence of POCI or Transcranial Doppler (TCD) that indicates vertebrobasilar insufficiency is needed. There will be no restrictions based on gender, age, race, and the course of the disease.

#### 3.1.3 Interventions

For experimental group, trials that use acupuncture therapy with or without conventional treatment or pharmacotherapy will be included; and acupuncture therapy involves: manual acupuncture (MA), electroacupuncture (EA), fire acupuncture (FA), warm acupuncture (WA), and scalp acupuncture (SA). For the corresponding control group, interventions could be placebo or waiting list control, sham-acupuncture, conventional treatment, or pharmacotherapy that consists of experimental group. Control group that uses acupuncture therapy will be excluded.

#### 3.1.4 Outcome indicators

##### 3.1.4.1 Primary outcomes

The primary outcome will include: Change of PCIV symptoms as well as the associated symptoms evaluated by different instrument including Dizziness Handicap Inventory, Vertigo Symptom Scale, University of California at Los Angeles dizziness questionnaire, and Vertigo Symptom Scale of traditional Chinese medicine;^[25,26]^ change of mean blood flow velocity (Vm), resistance index (RI), and pulsatility index (PI) of bilateral vertebral arteries (VA) and basilar artery tested by TCD. Measurement times are before the first intervention and after the last intervention.

##### 3.1.4.2 Secondary outcomes

The secondary outcomes will include: Barthel Index (BI), National Institute of Health Stroke Scale (NIHSS), clinical effectiveness, and adverse reactions. BI is used to evaluate the ability of daily living activity.^[27]^ NIHSS and clinical effectiveness are used to evaluate neurological function.^[28]^ Adverse reaction information will be involved to evaluate the safety of acupuncture therapy.

### 3.2 Data sources and search strategies

We will search articles in the following electronic databases: PubMed, EMBASE, Cochrane Library, Web of Science, the Chinese Biomedical Literature Database, the Chinese National Knowledge Infrastructure, and the Wan-fang databases and Chinese Scientific Journal Database (VIP) from inception to August 2020 with MeSH terms and key words, and without language restrictions. The search strategy is presented in Table 1.

**Table 1:**
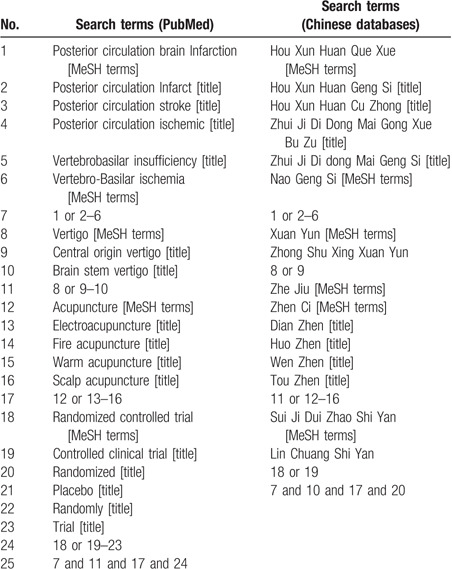
Search strategy.

References and conference literature will be searched manually to identify potential relevant studies. ClinicalTrials.gov and the International Clinical Trial Registry Platform will also be searched for completed and ongoing trials.

### 3.3 Study selection and data extraction

**Figure 1:**
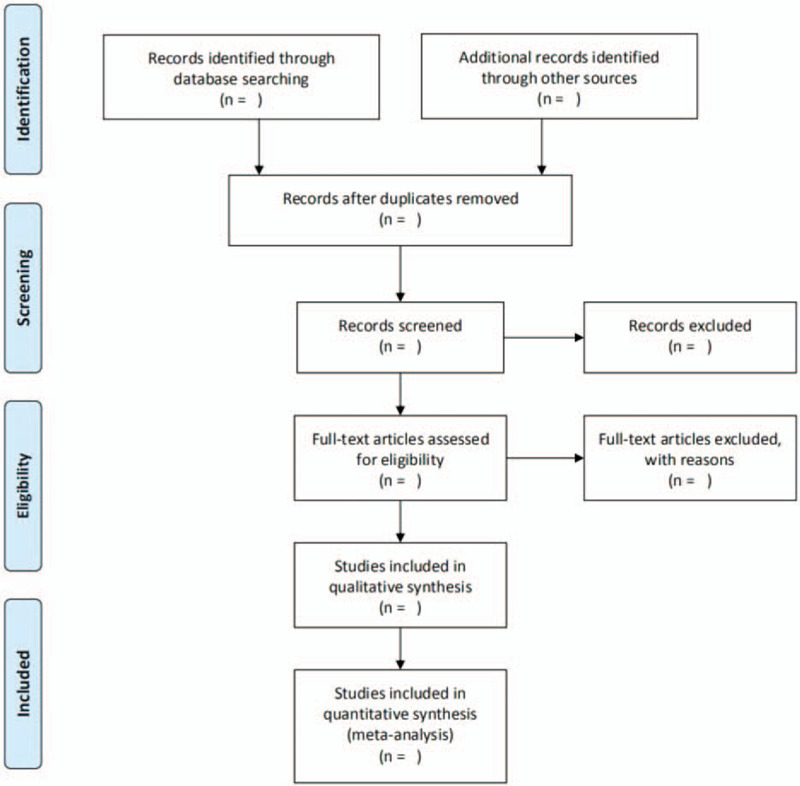
This picture reflects the steps of research selection, and explains the process of literature screening in detail.

Before data extraction, a standard data extraction form (EXCEL) will be proposed according to the specified indicators, and will be discussed among all the reviewers. After searching in the electronic database, we will import the result to Noteexpress software, version 3.2.0.(Aegean Sea software company Beijing, China). Then 2 reviewers will independently select studies. Duplicated researches and ineligible studies will be eliminated by reading title and abstract. Then other information will be confirmed by viewing the full text of article. After that, the following data of included studies will be extracted: title, language, first author, country, year of publication, number of participants, patient characteristics (age, gender, and disease course), study duration, funding source, interventions (type and time), randomization, allocation concealment method, blinding method, outcome indicators, follow-up duration, acupoint selection, and adverse events. Any disagreement will be resolved by discussion or by consulting a third reviewer until consensus is reached. Details of the selection process will be presented in the PRISMA flow chart (Fig. 1).

### 3.4 Methodological quality assessment and level of evidence

Two reviewers will use the updated Cochrane Risk of Bias Tool to assess the bias risk of the included trials.^[29]^ Any disagreement will be resolved by discussion or by consulting a third reviewer until consensus is reached. Each trial will be scored as high, low, or unclear risk for the following 7 domains: random sequence generation (selection bias); allocation concealment (selection bias); blinding of participants and personnel (performance bias); blinding of outcome assessment (detection bias); incomplete outcome data (attrition bias); selective reporting (reporting bias); and any other bias.

We will use Grading of Recommendations Assessment, Development, and Evaluation guidelines to assess the included evidence quality for primary outcomes,^[30]^ the result will be presented into 4 levels as high, moderate, low, or very low.

### 3.5 Statistical analysis

We will conduct statistical analysis using Review Manager (Revman) 5.3.0 (Cochrane Central Executive team, England) recommended by Cochrane Collaboration. For continuous variables (Vm, RI, PI, NIHSS, BI), the data will be presented data as mean, standard deviation, and median, and the result will be reported as mean difference or standard mean difference, with 95% confidence interval (CI). For dichotomous variables (clinical effectiveness and adverse events), the data will be presented by the numbers and proportions, and the result will be reported as relative risks with 95% CI.

### 3.6 Heterogeneity, subgroup analysis, and sensitivity

We will conduct heterogeneity analysis between trials using Revman software, with χ^2^ test and *I*^2^. For continuous variables, if significant heterogeneity is found (*P*<.05, *I*^2^>50%), random-effects model will be conducted for data synthesis, otherwise fixed-effects model will be used. For dichotomous variables, we will use random-effects model if the *P* value is less than.05. Subgroup analysis will be conducted for heterogeneity arising from interventions (MA, EA, FA, WA, and SA), control type (placebo or waiting list control, sham-acupuncture, conventional treatment, or pharmacotherapy), treatment duration, acupoints, and outcome indicators. Sensitivity analysis will be conducted in primary outcomes to test the result homogeneity. We will perform meta-analysis again after eliminating studies in low quality and will apply different statistical methods.

### 3.7 Assessment of publication bias

To assess publication bias risk arise from small sample size effect or study accuracy, a statistical analysis with funnel plot will be performed if more than 10 studies are included for meta-analysis.

### 3.8 Ethics and dissemination

This meta-analysis will not involve human beings, and all the information is published. Thus ethical approval will not be necessary. We will publish the results in a peer-reviewed journal.

## 4 Discussion

There is a long history for acupuncture treating PCIV, and clinical physicians have accumulated abundant experience over the 2000 years. In modern medicine, studies indicate that acupuncture can reduce discomforts of vertigo and decreases neurological impairment. A pilot study showed that acupuncture could improve cerebral blood flow velocity in poststroke patients,^[21]^ and animal experiment reveals that acupuncture effectively promoted cerebral perfusion of ischemic rats.^[31]^ Systematic and literature reviews have reported the beneficial effect of acupuncture treating stroke.^[32,33]^ While to our knowledge, no previous systematic review has focused on the effect of acupuncture treating PCIV. Considering that, we aim to assess the efficacy and safety of acupuncture for PCIV by searching comprehensive data sources and using systematic review and meta-analysis methods.

The result of this study will provide up-to-date evidence for the efficacy and safety of acupuncture in treating PCIV, which may help to establish an available option for clinicians and patients, as well as a reliable reference for further study.

## Author contributions

**Conceptualization:** Bo-xuan Li, Yu-zheng Du.

**Data curation:** Bo-xuan Li, Xiang-gang Meng, Chen Li.

**Formal analysis:** Xiang-gang Meng.

**Funding acquisition:** Chen Li.

**Investigation:** Bo-xuan Li.

**Methodology:** Xiang-gang Meng.

**Project administration:** Yu-zheng Du.

**Supervision:** Yu-zheng Du, Chen Li.

**Writing – original draft:** Bo-xuan Li.

**Writing – review & editing:** Yu-zheng Du.
